# Electro-Magnetism as a Means in the Treatment of Disease

**Published:** 1867-08

**Authors:** C. F. Hart

**Affiliations:** Chicago


### ELECTRO-MAGNETISM AS A MEANS IN THE TREATMENT OF DISEASE.

By C. F. Hart, M,D., Chicago.

In looking over the *Medical and Surgical Reporter* for June, 1867, No. 24, I find recorded three several cases treated by the galvanic battery. They were:—Case 1st, nævus of the eyelid, cured; Case 2d, papillary growth of the armpit, cured; Case 3d, moluscum of the right eyelid, cured, and without the usual cicatrix.

This brought to mind several cases treated by myself. The first was myself:—Some years ago, when a boy, about 16 years old, I recrived a hurt of the back, from lifting a log, which confined me to my bed for a week or ten days, and shortly after getting about, was thrown from my horse, over his head and against a log, the horse jumping on me with two feet on the small of my back. This again laid me up for some time, and for years after I suffered excruciating pains in the back and hips. I consulted many physicians, without relief, my sufferings growing. Finally, cystorrhœa supervened, with great pain and frequent hemorrhages in micturition; standing, walking, or hard riding would increase the pains. During this time, I had commenced the practice of medicine, and also commenced the investigation of my own case. As all internal remedies had failed, I concluded to try the electrolyptic apparatus, and, to my great joy and satisfaction, found relief in a short time. The mode adopted was placing one pole of the battery over the pubis, while the other was moved backwards and forwards across the lumbar region.

During the treatment of myself, a friend, and classmate, came into my office, suffering severely with neuralgia of the heart and scalp. He had tried almost everything, without more than temporary relief. I proposed to try the battery; he consented, and one pole was placed over the region of the heart, and the other to the head and neck, with great relief, the third or fourth application entirely succeeding. This was in 1859, and up to 1865 he had never been again troubled.

Again, whilst assistant-physician in the W. L. Asylum, in Kentucky, one of the male patients was suffering from incontinence of urine. I, in connection with the superintendent, had exhausted the various treatments of the books, without benefit, when I proposed to Dr. F. G. Montgomery to use the electrolyptic apparatus. He consented, and in less than a week there was a very decided improvement in his case, and in one month the patient was cured. Mode of applying—one pole over the pubis, the other in the perineum.

Another: During the great rebellion, an officer was wounded, at the battle of Shiloh, whilst in the act of giving a command, with his sword arm raised, the ball taking effect in the right breast, just under the clavicle and near the inner third, passing under the head of the humerus, through the axillary space, and making its exit near the elbow-joint, in its course severing some of the axillary plexus of nerves. He was taken home, and I waited on him during his recovery. In the mean time he complained a great deal of pain and burning in the palm of the hand, comparing it to a red-hot coal of fire laid there. One year afterwards he came home, with partial paralysis of the entire arm, with shrinking away of the muscles, and almost an entire loss of feeling in the hand. I tried the battery on him: the first time passing the to and fro current from the shoulder to the hand, for about ten minutes. The next evening it was repeated, and that night he had a return of the sensations during the healing of the wound and continued for several days, when I again used it on him. This time he complained considerable before leaving my office, and, the next day, told me he slept very little during the night in consequence of the pain, but that his sense of touch had very much improved, and he could use his arm much better. Here the treatment ended, not being able to persuade him to continue it.

I feel satisfied that had he continued the treatment, that it would have effected a cure.

Another, and the last, was an old physician, aged 73 or 74, with partial paralysis of the feet, or perhaps a more appropriate term would be, deficient innervation of the lower extremities, who used it with great benefit. Whether, or not, it ever produced a cure, I am unable to say, having left the place shortly after he commenced the treatment.

